# Wind Instrumentalists and Temporomandibular Disorder: From Diagnosis to Treatment

**DOI:** 10.3390/dj6030041

**Published:** 2018-08-23

**Authors:** Miguel Pais Clemente, Joaquim Mendes, André Moreira, Ricardo Vardasca, Afonso Pinhão Ferreira, José Manuel Amarante

**Affiliations:** 1 Faculty of Dental Medicine, University of Porto, 4200-393 Porto, Portugal; miguelpaisclemente@hotmail.com (M.P.C.); mimd12064@fmd.up.pt (A.M.); 2 INEGI-LAETA, Faculty of Engineering, University of Porto, 4200-465 Porto, Portugal; ricardo.vardasca@fe.up.pt; 3 Department of Orthodontics, Faculty of Dental Medicine, University of Porto, 4200-393 Porto, Portugal; aferreira@fmd.up.pt; 4 Department of Surgery, Faculty of Medicine, University of Porto, 4200-319 Porto, Portugal; amarante@med.up.pt

**Keywords:** wind instrumentalists, temporomandibular disorders, thermography, piezoresistive sensors, embouchure, temporomandibular joint biomechanics

## Abstract

Introduction: Temporomandibular disorders (TMD) involve the presence of pain or dysfunction on certain areas of the Cranio-Cervico-Mandibular Complex (CCMC), such as the masticatory muscles, the temporomandibular joint (TMJ) and associated structures like the postural muscles of the cervical region, can be considered as a sub-group of musculoskeletal disorders. Wind instrument players, as a consequence of their musical performance and its relation with the CCMC, can develop a TMD associated to muscle hyperactivity of certain elevator muscles, or even an increase of the intra-articular pressure in the functioning of the TMJ throughout musical activity. Aim: The objective of this paper is to describe the necessary and elementary steps in the diagnoses and treatment of a wind instrumentalist with a temporomandibular disorder, with the introduction of infrared thermography during this procedure. This case study also has the purpose of presenting the usefulness of piezoresistive sensors in the analysis of the clarinettists’ embouchure. Methodology: A Caucasian, 30-year-old female clarinettist was assessed through a clinical examination following the Diagnostic Criteria for TMD (RDC/TMD), as a complementary tool of diagnosis, a thermal imaging infrared camera, Flir E60 (Wilsonville, OR, USA), was used in order to analyse the above referred articular and muscular regions. The complementary examination protocol implemented with this clarinet player also involved the analyses of the embouchure with the support of piezoresistive sensors. Results: The clinical outcomes resulting from this work were based on the RDC/TMD diagnoses indicated that the clarinet player had an internal derangement on both TMJ, with an osteoarthritis on the left TMJ and an anterior disc displacement with reduction on the right TMJ. The infrared thermograms that were analysed, verified the existence of a temperature differential of the anterior temporal muscle (0.1 °C), the TMJ (0.1 °C) and the masseter muscle (0.7 °C), and after the occlusal splint therapy the asymmetry related to the master muscle reduced to 0.3 °C. The high pitches can reach values of 379 g of force induced to the tooth 21 comparing to the 88 g of force applied on tooth 11. The embouchure force measurements consistently presented greater forces during the higher notes, followed by the medium notes and finally the low notes and this happened with higher pressures being transmitted always to tooth 21. Conclusion: Performing arts medicine should understand the major importance of the dentistry field in the daily life of a professional musician, and the significance of implementing routine screening procedures of dental examinations, with infrared thermograms examination of distinct areas of the CCMC, as well as the use of sensors on the analyses of an eventual asymmetrical embouchure. Employing these techniques in dentistry will create the chance of preventing the overuse of some anatomical structures, with an early diagnosis and the correct monitoring of these areas.

## 1. Introduction

It is easy to understand that musicians are not typically viewed as having a dangerous profession; nevertheless, health practitioners are often unware or don’t realize how wind and string instrumentalists are exposed to many risk factors on a daily basis with high physical and psychological demands [1,2,3]. For such matter, it is important to value for example the complexity involved during the clarinet player’s embouchure that naturally occurs for an experienced musician, but takes time for a clarinet student to reach to perfection. In recent years, orofacial issues concerning musicians are being paid more attention, when usually the main focus in performing arts medicine was playing related musculoskeletal disorders [4,5,6]. This is interesting to take notice of, since temporomandibular disorders (TMD) which involved the presence of pain or dysfunction in certain areas of the Cranio-Cervico-Mandibular Complex (CCMC), such as the masticatory muscles, the temporomandibular joint (TMJ) and associated structures like the postural muscles of the cervical region, can be considered as a sub-group of musculoskeletal disorders [7].

Zaza et al. (1998), undertook a systematic review of published information on the incidence and prevalence of playing-related musculoskeletal disorders in classical musicians, being estimated that playing-related musculoskeletal disorders, to reach the prevalence of 39%, 47% in adults and 17% in secondary school music students [8]. Musculoskeletal disorders associated to these musicians can be reported to the area of the head and neck, where violin and viola players often report signs and symptoms identical to those of temporomandibular joint (TMJ) pain dysfunction syndrome. Zaza and Farewell studied different variables that could be associated with musician disorders: gender, instrument, body mass index and the number of years the musician has been playing [9]. The main risk factors that appeared in the study confirmed previous findings that female string players present a higher risk of playing related musculoskeletal disorders. Where the weight of the instrument and the overuse of certain muscles can be associated [8]. Playing-related musculoskeletal disorders have been studied by Zaza and Muszynski who identified 27 musicians that refer pain and other chronic symptoms that are beyond their control, and will interfere with their ability to play as usual [10]. The musicians normally seek medical treatment at a late stage because they have the belief “no pain, no gain”, so many of them think if there is any kind of pain, they are probably playing the correct way. The effort and hard work of practice is a natural routine, so if pain appears, it will be a consequence of many rehearsals.

Regarding wind instrument players, the consequence of their musical performance and its relation with the CCMC can be the appearance or development of a TMD, associated to muscle hyperactivity of certain elevator muscles, or even an increase of the intra-articular pressure in the functioning of the TMJ throughout musical activity. These conditions can occur associated to the implemented forces on the mouthpiece of wind instrumentalists, where his/her embouchure is intimately related with the TMJ biomechanics during the performance.

When a wind instrumentalist has a tooth rotation, there will be a natural response and adaptation of the embouchure. Curiously, one can say that the musician’s mouthpiece is the mirror of the instrumentalist’s embouchure, where a minimal and slight angulation in the anatomy of the incisal edge is sufficient to change the position of the clarinet. The research diagnostic criteria for TMD (RDC/TMD) is one of the most accepted diagnostic systems that is implemented with reliability in many epidemiologic and clinical studies of TMD. It uses operationally defined measurement criteria to generate computer-derived diagnostic algorithms for the most common TMD forms and provides specifications for conducting a standardized clinical physical examination [11,12]. The implementation of piezo-resistive sensors during the analyses of the wind instrument embouchure and infrared thermography to the correspondent zones of pain on the CCMC are certainly two important tools that can complement the analysis of the musician’s gesture [13,14]. Additionally, all of these matters mentioned above, emphasize the importance and the major role of a dentist in performing arts medicine.

In order to achieve a correct diagnosis, it is fundamental to understand the normal function of the stomatognathic apparatus and its relation with the instrument. The knowledge we have on the musical activity of the instrumentalist and the capacity of introducing biomedical techniques in order to monitor the performance of a wind instrumentalist, like in this case of a clarinet player, is essential for the final outcome of the treatment. Regarding the presence of a TMD on this single reed instrumentalist, there could be many options for the treatment. The different approaches for the treatment of a TMD are: cognitive behavioural therapy, acupuncture, physiotherapy, the use of an intra-oral appliances such as occlusal splints, pharmacologic treatment and in irreversible TMD pathologies surgical treatments [15,16,17,18,19,20,21,22,23].

Furthermore, it is important to bring to attention that the implementation of these different kinds of treatment regarding TMD has to do with the clinician's experience, the expectations of the patient and the will of the musician regarding the fact that they have to follow the treatment plan. At last, we should keep in mind that each individual will have its own biological response to the applied treatment. Independently to the type of treatment that is carried out, there is the possibility of monitoring any changes of the involved area and surrounding tissues. Within this perspective, infrared thermography can be a useful tool on quantifying the anatomo-physiology of specific regions of interest, prior and after the treatment of the TMD being implemented.

The objective of this paper is to describe the necessary and elementary steps in the diagnosis and treatment of a wind instrumentalist with a temporomandibular disorder, with the introduction of infrared thermography during this procedure. This case study also has the purpose of presenting the usefulness of piezoresistive sensors on the analyses of the clarinettists’ embouchure.

## 2. Methodology

**Figure 1:**
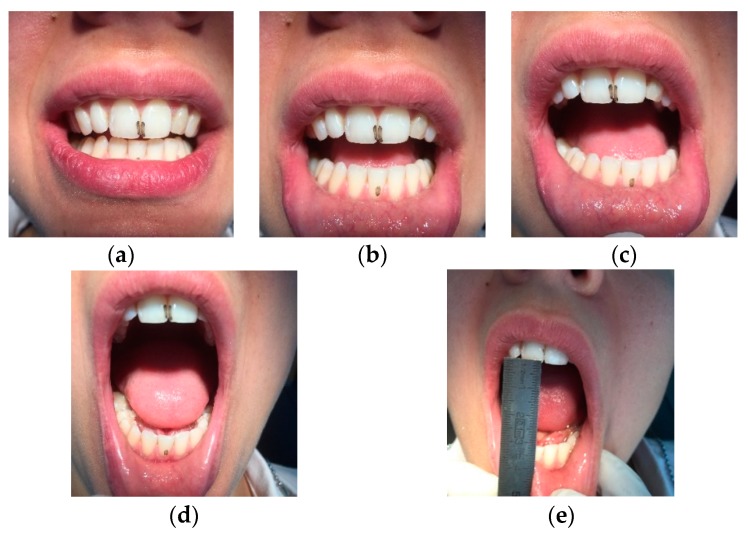
Analysis of the opening pattern of the patient and registration of the maximum pain free opening. (**a**) Middle line has 1 mm deviation to the left side in maximum intercuspation (IM); (**b**) During the condyle rotation the both jaws remain the same misalignment as in IM; (**c**) During the first part of condyle translation it can be seen a deviation to the left side; (**d**) Corrected deviation pattern to the left is confirmed; (**e**) 33 mm of maximum pain free opening.

**Figure 2:**
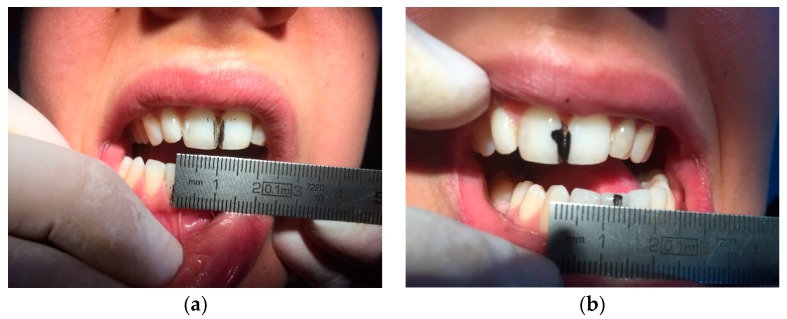
Analysis of the right and left eccentric movement and registration of the maximum range of motion. (**a**) Lateral right excursion of 11 mm; (**b**) Lateral left excursion of 12 mm.

**Figure 3:**
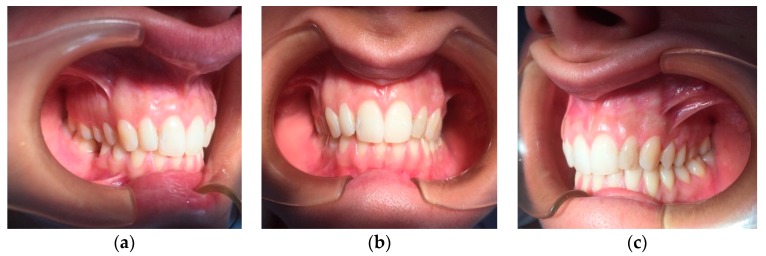
Occlusal analysis, (**a**) lateral right, (**b**) frontal and (**c**) lateral left pictures in maximum intercuspation.

A 30-year-old Caucasian clarinettist was assessed through a clinical examination following the Diagnostic Criteria for TMD (RDC/TMD), being previously questioned about: (a) the presence of pain in orofacial area or headaches in the last 30 days; (b) any sign of jaw joint noise, closed locking or open locking of the jaw; (c) the patient demographic information. The clinical examination of the clarinettist involved the evaluation of the mandibular cinematic with the opening patterns (Figure 1) that were assessed together with the lateral and protrusive movements (Figure 2), taking notes of the range of motion and areas with tenderness/pain during the maximum unassisted and maximum assisted opening of the mouth. Direct occlusal analysis was carried out providing data on static contacts between teeth in supportive areas, as well as on dynamic occlusal relations between the teeth—a type of laterotrusal guidance and interference contacts (Figure 3).

**Figure 4:**
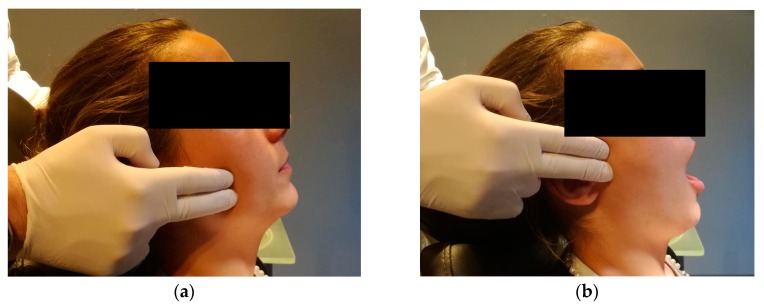
Extra-oral examination with the palpation of the masseter muscle and TMJ. (**a**) Masseter muscle palpation with the patient in rest position; (**b**) TMJ palpation during mouth opening/closing.

Concerning the extra-oral analysis, it was performed a bilateral palpation of the masseter and temporal muscles, and bilateral palpation of the TMJs during opening and closing movements, searching for the presence of tenderness/pain and noises, as well as during lateral and protrusive movements (Figure 4).

As a complementary tool of diagnosis, a thermal imaging infrared camera, E60 FLIR^®^, was used in order to analyse the above referred articular and muscular regions. This procedure was carried out before the clinical examination so that there wasn´t any kind of influence on the analysed areas from the pressure induced during the manual palpation. Prior to the thermographic examination, the clarinet player had a period of 15 min acclimatization within the dental office. Following the acquisition the thermal images, they were processed and examined using the software FLIR Tools v6.3, with the intent of quantifying the absolute temperature values of the regions corresponding to the anterior temporal muscle, superficial masseter muscle and TMJ.

**Figure 5:**
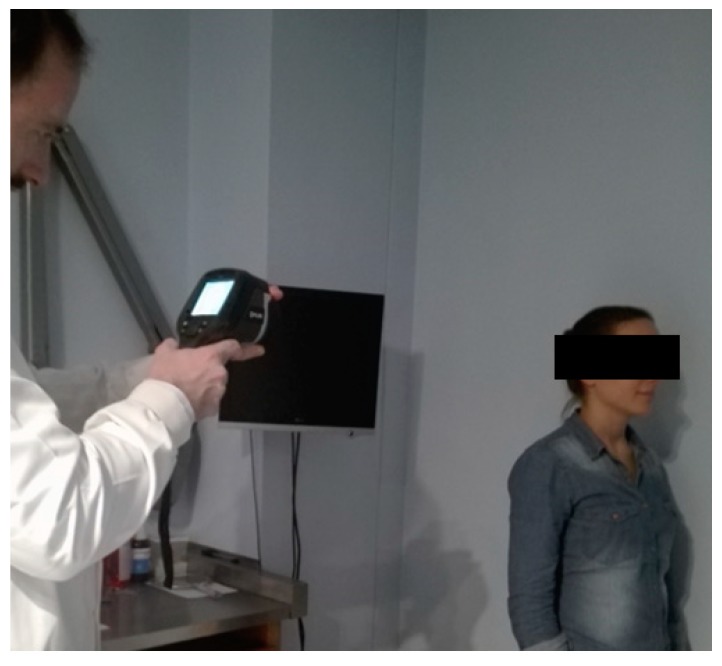
Recording the right lateral thermograms with the thermal imaging infrared camera, E60 FLIR.

The capture process of the infrared imaging requires rigorous protocol. Therefore, the following precautions regarding the thermographic examination were considered: (a) no coffee, alcohol, tobacco or drugs should be ingested prior to the exam; (b) no make-up, moisturizing cream or jewellery can be used; (c) no bath was taken at least one hour before the exam; (d) no physical exercise was made at least four hours before the exam; (e) the patient underwent the thermal images in a room without natural light, under temperature and humidity control; (f) the thermal camera was used and fixed at a distance of 1 m and a half from the single reed instrumentalist to obtain frontal, lateral right and left thermograms (Figure 5).

**Figure 6:**
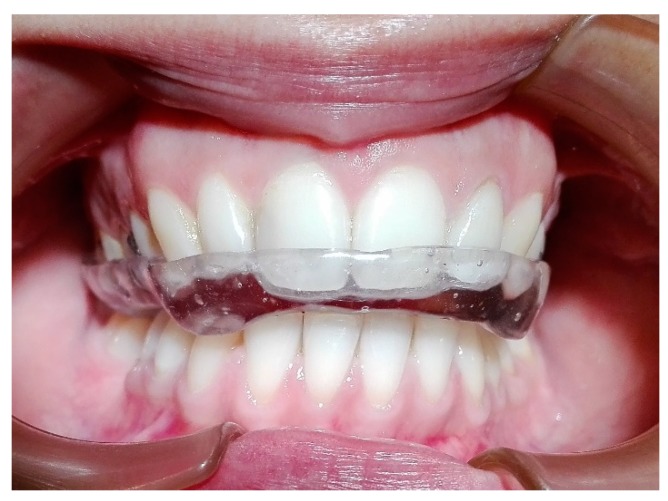
Occlusal splint, with uniform and bilateral dental contacts.

The clarinet player received a treatment plan based on the use of an occlusal splint (Figure 6). For the fabrication of the splint it was required the impression of both jaws, including all teeth alignment and surrounding tissues, a registration bite was performed in maximum intercuspation and the cast was mounted in a semi-adjustable articulator A7 plus with the corresponding facial arch. Posteriorly, the articulator was sent to the laboratory for the production of the acrylic splint. The occlusal splint is for the upper jaw, with a rigid acrylic full coverage of the occlusal surface. The splint has uniform and bilateral contacts, with multiples contact points for the mandibular cusps and incisal edges. A slight canine guidance was made in order to provide an interocclusal separation in the posterior zone during the excursion movements. The patient was counselled to wear the splint during the night whilst sleeping. A consultation was scheduled for calibration of the occlusal contacts after the first month. The patient attended another appointment after three months to check the occlusal splint and after six months there was an evaluation of the CCMC with a second thermographic examination.

**Figure 7:**
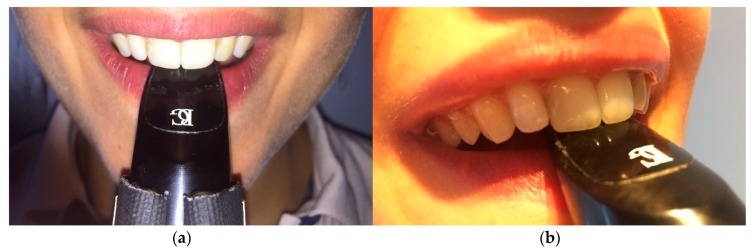
Analyses of the musician’s embouchure before incorporating the piezoresistive sensors in the mouthpiece. (**a**) The embouchure is slightly deviated to the left side where the tooth 21 contacts a large area of the mouthpiece; (**b**) The upper central incisors and the lower lip are responsible to stabilize the mouthpiece while performing the embouchure.

The complementary examination protocol implemented with this clarinet player also involved the analyses of the embouchure, conducted with the support of the piezoresistive sensors. A previous examination of the musician’s embouchure was performed, without the placement of any kind of sensors (Figure 7).

**Figure 8:**
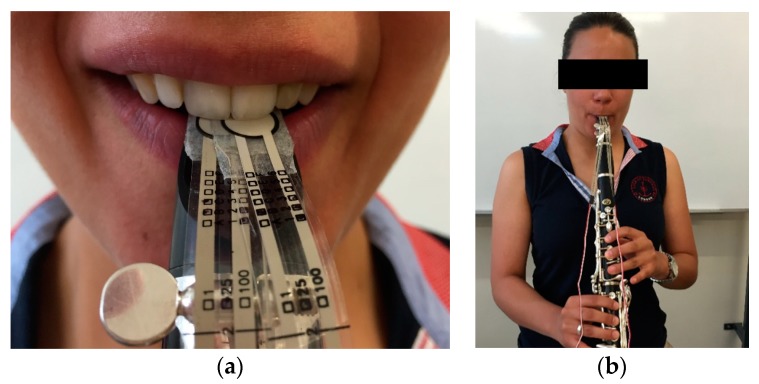
(**a**) Verifying the adaptation of the piezoresistive sensors on the musician’s mouthpiece; (**b**) Clarinet player performing different pitches whilst recording the pressure applied at the upper central incisors.

The clarinet player was encouraged to perform three different registration tones: high, medium and low. This procedure was repeated three times for each pitch (Figure 8), in order to fulfil the necessary criteria to obtain the median value of pressure.

## 3. Results

The clinical outcomes resulting from this work where the RDC/TMD diagnoses indicated that the clarinet player had an internal derangement on both TMJ, with an osteoarthritis on the left TMJ and an anterior disc displacement with reduction on the right TMJ. Concerning the muscular analyses, the results enabled the authors to check the presence of myofascial pain. In fact, these clinical observations are in agreement with the infrared thermograms that were analysed, since it was possible to compare the existing temperature differential of the areas assessed, namely the anterior temporal muscle with 0.1 °C, the TMJ with 0.1 °C and the masseter muscle with 0.7 °C (Table 1). These values correspond to the initial thermograms that were taken at the first appointment prior to the treatment with the stabilisation appliance.

**Figure 9:**
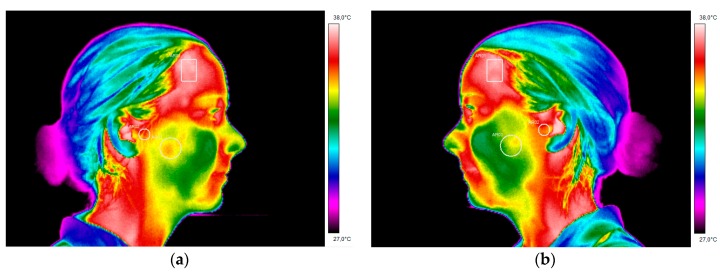
Right and left side infrared images of the clarinettist at the first appointment. (**a**) Right side thermogram, AR01—Region of interest corresponding to the temporal muscle, AR02—Region of interest corresponding to the TMJ, AR03—Region of interest corresponding to the masseter muscle; (**b**) Left side thermogram with the corresponding contralateral regions of interest being possible to observe the significant temperature differences in AR02.

**Figure 10:**
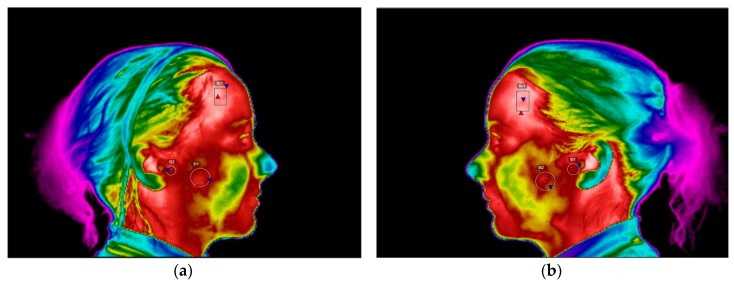
Right- and left- side infrared images of the patient at the end of the treatment. (**a**) Right side thermogram, Bx1 corresponds to the region of interest of the temporal muscle, EI02 corresponds to the region of interest of the TMJ, EI03 corresponds to the region of interest of the masseter muscle; (**b**) Left side thermograms with the contralateral corresponding regions of interest, where it is possible to observe a more symmetrical pattern.

It is possible to observe the region that corresponds to the masseter muscle shows lower temperature, on the right thermogram of the CCMC comparing to the contra-lateral region on the left thermogram, Figure 9. The temperature scale for Figure 9 and Figure 10 was set between 27 °C and 38 °C.

A second thermographic examination was performed six months subsequently to the first appointment and after the wind instrumentalist had been using the occlusal appliance. The regions of interest were analysed and compared with the temperature values obtained at the first appointment. The temperature differential of the masseter muscle decreased from 0.7 °C to the 0.3 °C, which means the occlusal splint allowed an equilibration and reduction in the muscular activity (Table 2).

The lateral right and left thermograms performed six months after wearing the occlusal splint presented more even temperature values on the masseter area and the TMJ. Nevertheless, there is still a presence of 0.3 °C temperature differential on the correspondent area of the masseter muscle (Figure 10).

Regarding the complementary examination protocol implemented with the support of the piezo-resistive sensors, it was possible to detect that the maximum pressure is being executed on tooth 21, reaching up to 408 g, comparing to the central incisor 11 with 82 g. The upper left central incisor always presents higher values of pressures in all of the different pitches (Table 3).

## 4. Discussions

To understand the usefulness of infrared thermography as a complementary tool during the clinical examination of the clarinet player, it is fundamental to understand the biomechanics of the temporomandibular joint. The patient underwent a meticulous intra-oral and extra-oral observation with palpation of the temporomandibular joint and the masticatory muscles, in particular the masseter and temporal muscles. The clinical examination followed the analysis of the mandibular cinematic, being able to observe the opening pattern with a deviation to the left side and then returning to the midline. This was coincident to the main symptoms referred by the clarinettist of the existing pain in the left temporomandibular joint. This factor is relevant since it is known that in the presence of an internal derangement, the temporomandibular joint can present a delay in the translation of the condyle with the disc, which will promote a deviation to the side of the affected TMJ.

This was precisely what happened with this clarinet player and has direct implications on the wind instrumentalist embouchure, like it will be discussed hereafter. The mouthpiece is placed inside the musician’s mouth, in this case in order to centre her embouchure it would be necessary to have a regular opening of the mouth where both temporomandibular joints would perform a symmetrical and smooth movement, even within an amplitude of 20–30 mm. Nevertheless, what happened during the beginning of the mouth opening is that the TMJ promotes a rotation of the condyle and the disc, where the clarinet was stabilized with the mouthpiece between the upper central incisors and the lower lip that is retruded over the lower incisors. This will happen at the best “convenience” position adopted by the mandible after opening the mouth and where there will be the least effort for the orofacial structures involved. In practice, what happens is that the musician will adapt the mouthpiece towards the side of the “most” affected TMJ, since there is a reduced movement of the affected TMJ. Therefore, what will occur is that the clarinettist, in order to allow a centred embouchure regarding the position of the upper central incisors, will have a difference in the movement of the condyle-disc complex throughout the slope of the eminence of the mandibular fossa. The displacement of the condyles down the slope will involve the contraction of the lateral pterygoid muscles, since this muscle is in activity when pulling one or both condyles forward and downward. Simultaneously there is an isometric contraction of the masseter muscle, when stabilizing the mouthpiece inside the mouth. Since the biomechanics of the TMJ of this wind instrumentalist is modified, as it was possible to assess during the palpation of the TMJ during the clinical examination, there will also be an uncoordinated muscle action of certain muscle fibres, like the superficial masseter. This could be observed after analysing the musicians thermograms, that permitted the measurement of the existing asymmetry, by the temperature value of 0.7 °C, on the area corresponding to the masseter muscle. To our knowledge, this is in accordance to a higher muscle activity, and consequent hyperactivity of the right masseter muscle, when comparing with the contralateral side. In the past, other studies have characterized the thermal patterns of individuals with TMD, having the differential value of 0.3 °C as a reference for the asymmetry temperature that could indicate the presence of discomfort/pain of the affected area when comparing to the contralateral side [24,25].

From a clinical point of view, this reflects the slight deviation of the mandible to the left side, and the essential compensation of the right masseter muscle, whose superficial fibres also have the purpose of promoting the protrusion of the mandible. So even if there is a centred embouchure from the point of view of the mouthpiece being aligned with the upper central incisors, the veracity of this fact can eventually not correspond to the exact movement executed by the condyles, as they rotate and slide forward down the eminence taking in consideration the initial position of the condyle at a most superior position.

Following the understanding of the fundamental principles involved during the biomechanics of the TMJ of a clarinet player, in this particular case has a temporomandibular disorder with the presence of discomfort/pain on the left TMJ and right masseter, it was possible to correlate these symptoms with the existence of muscle hyperactivity on the right masseter muscle. With these facts, we have a third crucial point for analysis regarding the musician´s performance, which is the anterior zone of the maxilla and the incisal edge of the upper central incisors where the forces of the embouchure are transmitted during the “grip” of the mouthpiece. For this matter, taking into consideration the results of the piezo-resistive sensors it was possible to observe another important parameter during the single reed instrumentalist’s performance, the embouchure force measurement demonstrated an asymmetrical force distribution between teeth 11 and 21. During the embouchure and subsequent musical performance, tooth 21 is being the subject of a higher pressure than tooth 11 and this phenomena occurs in every range of pitch. The high pitches can reach values of 379 g of force induced to the tooth 21 comparing to the 88 g of force applied on tooth 11. The embouchure force measurements, presented always greater forces during the higher notes, followed by the medium notes and finally the low notes and this happened with higher pressures being transmitted always to tooth 21.

One of the possible interpretations may be due to the fact that it is more convenient and comfortable for the clarinet player to stabilize the mouthpiece employing a higher pressure on tooth 21, since there can be a slighter movement of the mandible to the left side of the musician. This deviation related to the internal derangement of the left TMJ, will involve a higher activity of the right masseter muscle. As confirmed in the infrared thermogram, the activity of this strong elevator muscle will promote a higher impact and pressure on the contralateral central incisor, the tooth 21. The forces can reach up to values four times higher on the central incisor 21 comparing with the central incisor 11.

Altenmuller et al. in the past already attempted to analyse and study the different movements of the mandible during the musicians’ embouchures [26]. This examination was performed in brass instrumentalists, using MRI, but there are certain limitations that this imagiology process brings regarding the musicians embouchure. The main, and principle difference, was the mouthpiece of the brass instrument players was changed to a no metal material and the second reason, also with major importance, if not the main reason for some limitations of the study, was that the musicians were lying down within the MRI system [27]. This issue will not allow the understanding of the biomechanics of the TMJ, in the most similar conditions as when the wind instrumentalists are performing their embouchure. On the other hand, one of the most effective procedures to analyse the position of the TMJ during musical performance would be a computed axial tomography of the TMJ, with the mouthpiece placed on the mouth, and eventually understand the differences of the adopted position of the TMJ on the mandibular fossa. Nevertheless, this procedure would oblige a higher and unnecessary risk of X-ray exposition to the musician. An alternative could be the use of an ARCUSdigma^®^ device from Kavo, Biberach an der Rib, Germany, in order to study the condylar movement pathway for each TMJ, but the employment of this technique would only be suitable for the analyses without the mouthpiece. This procedure with the introduction of the mouthpiece in the musician’s mouth is not reasonable, since the ARCUSdigma^®^ has a clench that is adapted to the upper teeth, which will not allow the correct position of the mouthpiece. So, according to the authors’ opinion, this visualization of the mouthpiece in relation with the orofacial structures could be made with a lateral cephalogram, that can be an efficient method for a correct interpretation of the wind instrumentalist’s embouchure, by a minimum X-ray exposure. To complement this procedure, in this article it was described the application of infrared thermography and the piezo-resistive sensors which were fundamental to characterize the anatomo-physiological processes involved in the clarinet player’s embouchure.

This patient underwent occlusal splint therapy in order to reduce the symptomatology associated to the TMD and was also educated about cognitive behavioural changes. Regarding the existing asymmetry pattern of the TMJ from 0.1 °C to 0 °C, this can be difficult for interpretation since when both TMJ are affected, they can be both pathological and it is not possible to claim from the analysis of a thermogram that there is a TMJ from one side more affected than the other. Exactly like in this particular case where both present an internal derangement, with osteoarthritis on the left TMJ and an anterior disc displacement with reduction on the right TMJ. In this case, the use of infrared imaging was useful as a complementary method of diagnosis and treatment, since it was possible to observe the differences of the lateral thermograms especially in the region of interest of the masseter muscle where there was a reduction on the differential temperature from 0.7 °C to 0.3 °C. This can be related to the use of the occlusal splint at night, which is in accordance to an improvement in the occlusal stability and a reduction in muscle activity during the parafunctional habit of bruxism during the sleeping periods. The use of this intraoral device can be also associated to the reestablishment of a proper occlusal relationship which will be favourable for the reduction of the deleterious muscle hyperactivity [18,28,29]. This leads to a decrease of the masticatory muscle pain and discomfort felt on the TMJ.

In general, the analysed parameters reported significant improvement from the first appointment, to six months after the implemented treatment using the occlusal splint. These significant improvements are referred mainly to the myofascial pain present on the masseter muscle, since the articular pathology affecting the TMJ with osteoarthritis in the left TMJ and anterior displacement with reduction on the right TMJ will still be present but with a decrease in the symptomatology. In fact, the infrared thermography gives the chance of quantifying these variations and allows clinicians to complement their diagnoses not depending only on subjective evaluations. Nevertheless, the thermograms regarding the region of interest of the TMJs showed a minor decrease of 0.1 °C from one appointment to the other, which is in accordance to this circumstance where both TMJs are pathological. Consequently, it is difficult to compare the thermographic values of these specific areas when both TMJs are affected, leaving to the clinicians the capacity of verifying during the clinical examination the evolution of the implemented treatment. The analysis of the CCMC with infrared thermography was standardized through the usage of a thermal imaging capture protocol based in the internationally accepted guidelines where the differences between the skin temperature are explained by the underlying physiology, which can represent pathological states [30,31,32,33].

Therefore, it is important to highlight the advantages of techniques such as infrared thermography and the piezoresistive sensors as a complementary method on the analysis of the CCMC of a wind instrumentalist, where the differential diagnostics of TMD is based on a standardized clinical examination. However, if dentists can use biomedical devices to complement their activity they can probably achieve better results, from diagnosis to treatment.

## 5. Conclusions

The diagnoses of TMD is a challenging field, since its etiology is multifactorial. When leading with wind instrumentalists this task is even more difficult since the main option of trying to eliminate a predisposing factor, such as playing the clarinet, it is not a solution. For a professional musician it is crucial that the dentist may be able to understand the interrelations of the musician’s embouchure, where the mouthpiece, the temporomandibular joint, the elevator muscles and the upper central incisors become a unique structure and the variation in one of these anatomic areas will directly influence the other.

Being able to understand the biomechanics of the temporomandibular joint and having the necessary resources in terms of biomedical devices, like infrared thermography and sensors, can be an added value when diagnosing and treating a wind instrumentalist with a TMD. The use of an occlusion splint during the night was adequate to reduce the TMD symptomatology present on the clarinet player.

Performing arts medicine, should understand the major importance of the dentistry field in the everyday of a professional musician, and the significance of implementing routine screening procedures of dental examinations, with infrared thermograms examination of distinct areas of the CCMC, besides the use of sensors on the analysis of an eventual asymmetrical embouchure. Employing these techniques in dentistry will create the chance of preventing the overuse of some anatomical structures, with an early diagnosis and a correct monitoring of these areas.

## 6. Patents

No patents may or will result from this work.

## Figures and Tables

**Table 1:** Infrared imaging values of the thermic difference between left- and right-side at the first appointment.

Areas Assessed	Degrees of Temperature Asymmetry (°)
Temporalis	0.1
Temporomandibular Joint	0.1
Masseter	0.7

**Table 2:** Infrared imaging values of the thermic difference between left- and right-side at the end of the treatment.

Areas Assessed	Degrees of Temperature Asymmetry (°)
Temporalis	0.1
Temporomandibular Joint	0.0
Masseter	0.3

**Table 3:** Piezo-resistive sensors’ results of three different pitches; high, medium and low, registered at the upper central incisors during the embouchure mechanism.

Pitch	Trial Number	Average (g)		Standard Deviation	
		Tooth 1.1	Tooth 2.1	Tooth 1.1	Tooth 2.1
High	Trial 1	0.089	0.375	0.014	0.044
	Trial 2	0.094	0.354	0.016	0.027
	Trial 3	0.082	0.408	0.017	0.038
Medium	Trial 1	0.035	0.274	0.009	0.039
	Trial 2	0.031	0.296	0.041	0.039
	Trial 3	0.040	0.295	0.005	0.022
Low	Trial 1	0.031	0.242	0.014	0.025
	Trial 2	0.045	0.222	0.014	0.051
	Trial 3	0.031	0.267	0.006	0.021
